# Acupuncture for diabetic neuropathic pain: A protocol for systematic review and meta analysis

**DOI:** 10.1097/MD.0000000000023244

**Published:** 2020-11-20

**Authors:** Liqin Wang, Zhaohong Gao, Xiangru Niu, Meiqi Yuan, Yan Li, Fei Wang, Chuang Guo, Zhen Ren

**Affiliations:** a Department of First Clinical Medicine,First Affiliated Hospital of Heilongjiang University of Chinese Medicine; b Department of Graduate school, Heilongjiang University of Chinese Medicine, Harbin,China.

**Keywords:** acupuncture, diabetic neuropathic pain, meta-analysis, systematic review

## Abstract

**Background:**

Diabetic neuropathic pain (DNP) is a common complication of diabetes mellitus, it severely affects the quality of life of Diabetic patients. Acupuncture is proofed to have favorable effects in treating DNP, however, evidence needs to be gathered and interpreted. We will make a comprehensive review of clinical trials concerning acupuncture in treating DNP and do meta-analysis if possible.

**Method:**

The following databases will be searched from the inception to September 2020: PubMed, Embase, Web of Science, China National Knowledge Infrastructure, Wan-Fang Database, and Chinese Scientific Journal Database. RCTs that evaluated acupuncture for patients with DNP will be included. The primary outcome will be patient-reported pain intensity using validated scales or verbal reporting. The secondary outcomes including the Toronto clinical scoring system, Sensory Nerve Conduction Velocity, Motor Nerve Conduction Velocity, and quality of life. The study selection, data extraction, and study quality evaluation will be performed independently by 2 researchers. A meta-analysis will be performed using RevMan V5.3 statistical software if possible; otherwise, descriptive analysis or subgroup analysis will be conducted. The quality of evidence for outcomes will be assessed with the Grading of Recommendations Assessment, Development and Evaluation (GRADE) approach.

**Results:**

This study will evaluate the effect and safety of acupuncture in treating DNP.

**Conclusions:**

The evidence we generated from the present study will provide more options for DNP management in clinical practice.

**Systematic review registration:**

INPLASY202090043.

## 1 Introduction

Diabetic neuropathic pain (DNP) is one of the most common complications of diabetes mellitus, affecting approximately 30%-50% of diabetes patients.^[1–3]^ The main symptoms of DNP include spontaneous pain and irritation-induced pain.^[4,5]^ Spontaneous pain can be manifested as constant burning, sharp, shooting, or even as electric shock sensations. Stimulus-induced pain includes hyperalgesia and allodynia. Hyperalgesia refers to stimuli that can cause pain under normal conditions, and the pain is more severe than normal; allodynia refers to stimuli that do not cause pain under normal conditions (such as tactile) leads to pain. DNP is usually considered moderate to severe and often worse at night, causing sleeping disturbs.^[4,5]^ The pain can be last for years, restrict daily activity, and affect the quality of life.^[6]^

DNP is hard to treat. Its management and treatment require a series of approaches including early recognition, glycemic control, psychological therapy, and agents for symptomatic pain relief.^[7]^ Oral treatment is most commonly used as pain relief; however, it is associated with the risk of adverse effects since some DNP patients also suffer from multiple comorbidities and altered pharmacokinetics and pharmacodynamics that may alter drug metabolism. Also, pain reliefs currently used such as duloxetine, pregabalin, tapentado, and norepinephrine are unsatisfied for most DNP patients, new managements for DNP are needed.^[8,9]^

Complementary and alternative medicine interventions are being used to treat DNP. Acupuncture and electroacupuncture are proved to be effective in pain management in many conditions such as persistent tissue injury (inflammatory), nerve injury (neuropathic), cancer, and visceral pain, and they could also forestall the side effects of often debilitating pharmaceuticals.^[10]^

There are several small sample size randomized controlled trials concerning acupuncture in treating DNP, however, to the best of our knowledge, there is no systematic review to summaries the results.^[11–13]^ Therefore, a comprehensive review of acupuncture in treating DNP is needed, and the evidence could provide more options for DNP management.

## 2 Methods

### 2.1 Study registration

This systematic review protocol has been registered on INPLASY. The registration number was INPLASY202090043. The protocol followed the Preferred Reporting Items for Systematic Reviews and Meta-Analysis Protocol (PRISMA-P) statement guidelines.^[14]^

### 2.2 Eligibility criteria

#### 2.2.1 Types of studies

We will include all randomized controlled trials (RCTs) regarding acupuncture in treating DNP. Nonrandomized clinical studies, cluster randomized trials, and quasi-randomized trials will be excluded. The language is limited to English and Chinese.

#### 2.2.2 Types of participants

Patients diagnosed with DNP will be included regardless of sex, age, race, education, and economic status.

#### 2.2.3 Type of interventions

Acupuncture is defined as needle stimulation of acupoints, we will include studies using body acupuncture, scalp acupuncture, manual acupuncture, auricular acupuncture, electro-acupuncture, fire needling et al. Studies using acupressure, moxibustion, laser acupuncture, pharmaco-acupuncture, transcutaneous electrical nerve stimulation will be excluded. Studies regarding acupuncture as adjunctive treatment compared with other treatments will also be included.

Control intervention will include any other treatments used to manage DNP other than acupuncture.

#### 2.2.4 Types of outcome measures

The primary outcome measure will be patient-reported pain intensity using validated scales (e.g., visual analogue scales (VAS), numerical rating scales), or verbal reporting. The secondary outcomes including the Toronto clinical scoring system (TCSS), Sensory Nerve Conduction Velocity(SNCV), Motor Nerve Conduction Velocity(MNCV), and quality of life(36-Item Short Form Health Survey (SF-36)).

### 2.3 Search strategy

The reviewers will conduct a systematic literature search in the following electronic databases: PubMed, Embase, Web of Science, China National Knowledge Infrastructure (CNKI), Wan-Fang Database and Chinese Scientific Journal Database (VIP database). The search dates will be set from the inception to September 2020. The sample of the search strategy for PUBMED is presented in Table 1.

**Table 1:** Search strategy.

Number	Search items
1	Acupuncture
2	Acupuncture therapy
3	Electroacupuncture
4	Auricular acupuncture
5	Needling
6	Acupoints
7	Moxbustion
8	1 or2–7
9	Diabetic neuropathies
10	Autonomic Neuropathies, Diabetic
11	Diabetic Neuralgias
12	Diabetic Neuropathy, Painful
13	Symmetric Diabetic Proximal Motor Neuropathy
14	Diabetic Asymmetric Polyneuropathy
15	Polyneuropathies, Diabetic
16	9 or 10–15
17	Randomized controlled trial
18	Randomized
19	Randomly
20	Clinical trial
21	17 or 18–20
22	8 and 16 and 21

### 2.4 Data selection and extraction

#### 2.4.1 Study selection

**Figure 1:**
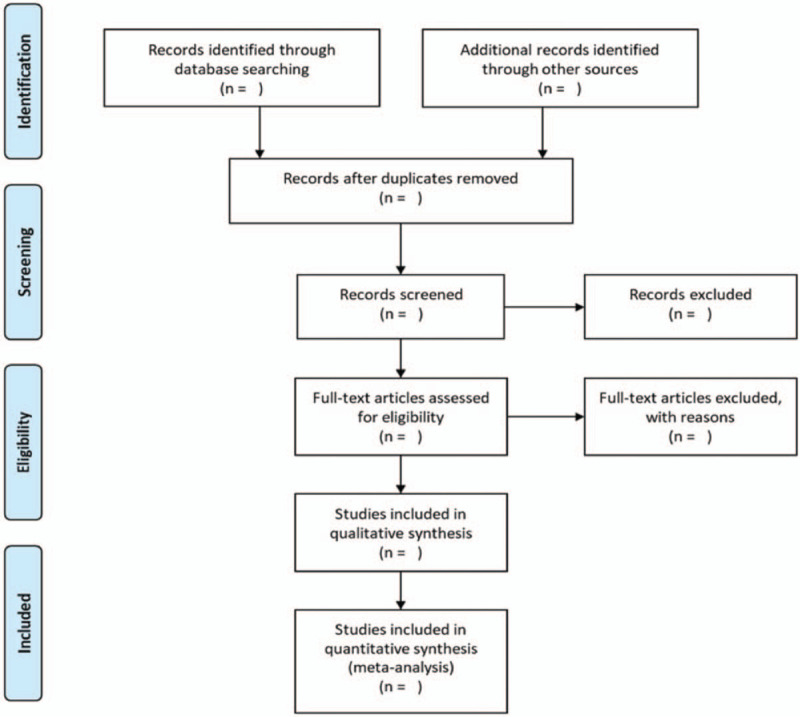
Flow chart of study selection.

Paired investigators (ZG and XN) will independently screen all titles and abstracts to get qualified studies, and then excluded duplications. After that, the full text of all potential studies will be checked for further screening. We will record all removed studies with specific reasons. Discrepancies will be resolved via referencing the original article and via group discussions or in consultation with the principal investigator (LW). The whole process of study selection is summarized as a flowchart in Figure 1.

#### 2.4.2 Data extraction

Two independent investigators (ZG and MY) will extract and tabulated all data using a standardized data extraction form. Discrepancies will be resolved via referencing the original article and in consultation with the principal investigator (LW).

The following data will be extracted including leading author, year of publication, journal, country or region, study design, sample size, patients age, diagnostic criteria of DNP, type of intervention, controls, treatment frequency, outcome measures, adverse effects, and any other relevant information.

#### 2.4.3 Assessment of risk of bias in included studies

The risk of bias of included study will be assessed using the Cochrane collaborative tool. We will evaluate the following aspects of the studies including sequence generation, assignment sequence hiding, blindness of participants and staff, outcome evaluators, incomplete result data, selective result reporting, and other sources of bias. The risk of bias is evaluated at 3 levels, namely, low risk, high risk, and ambiguity. If the information is not clear enough, we will try to contact the author of the article for further information.

### 2.5 Statistical analysis

We will use Review Manager Software (RevMan) V.5.3 for data synthesis, meta-analysis. Mean difference or standardized mean difference and 95% confidence intervals (CIs) will be used to calculate quantitative data, and dichotomous data will be exerted as risk ratio and 95% CIs. Statistical heterogeneity across studies will be done with *I*^2^ statistic. *I*^2^ ≤ 50 indicates homogeneity among studies, and a fixed-effects model will be employed for pooled analysis. *I*^2^ > 50% suggests obvious heterogeneity, and a random-effects model will be employed for synthesized analysis.

When there is homogeneity of the merged outcome results across sufficient studies, meta-analysis will be conducted.

### 2.6 Subgroup analysis

Subgroup analysis will be conducted based on the difference of interventions, controls, outcome measurements, and so on if necessary.

### 2.7 Sensitivity analysis

Sensitivity analysis will be undertaken to check the stability of merged outcome results by excluding studies with high risk of bias if significant heterogeneity exists.

### 2.8 Quality of evidence

The quality of evidence for outcomes will be assessed with the Grading of Recommendations Assessment, Development and Evaluation (GRADE) approach.^[15]^

The evaluation included bias risk; heterogeneity; indirectness; imprecision; publication bias. And each level of evidence will be made as very low, low, moderate, and high.

## 3 Ethics and dissemination

Since this is a protocol of systematic review and meta-analysis, ethics approval is not required. We will report our findings of this systematic review and meta-analysis in a peer-reviewed journal in the future.

## Author contributions

**Conceptualization:** Liqin Wang, Yan Li, Fei Wang, Chuang Guo, Zhen Ren.

**Data curation:** Zhaohong Gao, Xiangru Niu, Meiqi Yuan.

**Formal analysis:** Zhaohong Gao, Xiangru Niu, Meiqi Yuan.

**Funding acquisition:** Liqin Wang.

**Methodology:** Yan Li.

**Project administration:** Liqin Wang.

**Writing – original draft:** Yan Li.

**Writing – review & editing:** Liqin Wang, Zhaohong Gao, Yan Li.

## References

[1] Spallone V Lacerenza M Rossi A Painful diabetic polyneuropathy: approach to diagnosis and management. Clin J Pain 2012;28:726–43. 22209797 10.1097/AJP.0b013e318243075c

[2] Snyder MJ Gibbs LM Lindsay TJ Treating painful diabetic peripheral neuropathy: an update. Am Fam Physician 2016;94:227–34. 27479625

[3] Griebeler ML Morey-Vargas OL Brito JP Pharmacologic interventions for painful diabetic neuropathy: an umbrella systematic review and comparative effectiveness network meta-analysis. Ann Intern Med 2014;161:639–49. 25364885 10.7326/M14-0511

[4] Schreiber AK Nones CF Reis RC Diabetic neuropathic pain: Physiopathology and treatment. World J Diabetes 2015;6:432–44. 25897354 10.4239/wjd.v6.i3.432 PMC4398900

[5] Yang XD Fang PF Xiang DX Topical treatments for diabetic neuropathic pain. Exp Ther Med 2019;17:1963–76. 30783472 10.3892/etm.2019.7173 PMC6364237

[6] van Hecke O Austin SK Khan RA Neuropathic pain in the general population: a systematic review of epidemiological studies. Pain 2014;155:654–62. 24291734 10.1016/j.pain.2013.11.013

[7] Ziegler D Painful diabetic neuropathy: treatment and future aspects. Diabetes Metab Res Rev 2008;24: Suppl 1: S52–7. 18395890 10.1002/dmrr.817

[8] Kaku M Vinik A Simpson DM Pathways in the diagnosis and management of diabetic polyneuropathy. Curr Diab Rep 2015;15:609. 25899758 10.1007/s11892-015-0609-2 PMC4440893

[9] Baron R Förster M Binder A Subgrouping of patients with neuropathic pain according to pain-related sensory abnormalities: a first step to a stratified treatment approach. Lancet Neurol 2012;11:999–1005. 23079556 10.1016/S1474-4422(12)70189-8

[10] Zhang R Lao L Ren K Mechanisms of acupuncture- electroacupuncture on persistent pain. Anesthesiology 2014;120:482–503. 24322588 10.1097/ALN.0000000000000101 PMC3947586

[11] Chao MT Schillinger D Nguyen U A Randomized clinical trial of group acupuncture for painful diabetic neuropathy among diverse safety net patients. Pain Med 2019;20:2292–302. 31127837 10.1093/pm/pnz117 PMC7963203

[12] Shin KM Lee S Lee EY Electroacupuncture for painful diabetic peripheral neuropathy: a multicenter, randomized, assessor-blinded, controlled trial. Diabetes Care 2018;41:e141–2. 30061320 10.2337/dc18-1254

[13] Ahn AC Bennani T Freeman R Two styles of acupuncture for treating painful diabetic neuropathy--a pilot randomised control trial. Acupunct Med 2007;25:11–7. 17641562 10.1136/aim.25.1-2.11

[14] Shamseer L Moher D Clarke M Preferred reporting items for systematic review and meta-analysis protocols (PRISMA-P) 2015: elaboration and explanation. BMJ 2015;350:g7647. 25555855 10.1136/bmj.g7647

[15] Balshem H Helfand M Schünemann HJ GRADE guidelines: 3. Rating the quality of evidence. J Clin Epidemiol 2011;64:401–6. 21208779 10.1016/j.jclinepi.2010.07.015

